# Preventative and Therapeutic Effects of Astaxanthin on NAFLD

**DOI:** 10.3390/antiox12081552

**Published:** 2023-08-03

**Authors:** Nor Hafiza Sayuti, Khairul Najmi Muhammad Nawawi, Jo Aan Goon, Norfilza Mohd Mokhtar, Suzana Makpol, Jen Kit Tan

**Affiliations:** 1 Department of Biochemistry, Faculty of Medicine, Universiti Kebangsaan Malaysia, Bandar Tun Razak, Cheras, Kuala Lumpur 56000, Malaysia; norhafizasayuti@ukm.edu.my (N.H.S.);; 2 Gastroenterology and Hepatology Unit, Department of Medicine, Faculty of Medicine, Universiti Kebangsaan Malaysia, Bandar Tun Razak, Cheras, Kuala Lumpur 56000, Malaysia; 3 GUT Research Group, Faculty of Medicine, Universiti Kebangsaan Malaysia, Bandar Tun Razak, Cheras, Kuala Lumpur 56000, Malaysia; 4 Department of Physiology, Faculty of Medicine, Universiti Kebangsaan Malaysia, Bandar Tun Razak, Cheras, Kuala Lumpur 56000, Malaysia

**Keywords:** non-alcoholic fatty liver disease, NAFLD, non-alcoholic steatohepatitis, NASH, astaxanthin, carotenoid, liver disease, steatosis, antioxidant

## Abstract

Non-alcoholic fatty liver disease (NAFLD) is a significant public health issue owing to its high incidence and consequences, and its global prevalence is presently 30% and rising, necessitating immediate action. Given the current controversies related to NAFLD, the search for novel therapeutic interventions continues. Astaxanthin is a carotenoid that primarily originates from marine organisms. It is the best antioxidant among carotenoids and one of the most significant components in treating NAFLD. The use of astaxanthin, a xanthophyll carotenoid, as a dietary supplement to treat chronic metabolic diseases is becoming more evident. According to growing data, astaxanthin may be able to prevent or even reverse NAFLD by reducing oxidative stress, inflammation, insulin resistance, lipid metabolism, and fibrosis. Astaxanthin might become a viable therapeutic or treatment option for NAFLD in the upcoming years. Elucidating the impact and mechanism of astaxanthin on NAFLD would not only establish a scientific basis for its clinical application, but also potentially enhance the precision of experimental methodology for future investigations targeting NAFLD treatment. This review explores the potential preventive and therapeutic effects of astaxanthin on liver disorders, especially NAFLD.

## 1. Introduction

Non-alcoholic fatty liver disease (NAFLD) is a clinicopathological illness characterised by excessive fat accumulation in hepatocytes, excluding alcohol and other particular liver-damaging agents. NAFLD is expected to take on a predominant role as the leading aetiology of advanced liver disease in the forthcoming decades [1]. NAFLD is a condition that progresses from simple fatty liver (steatosis) to a severe form of non-alcoholic steatohepatitis (NASH) [2], which can result in irreparable organ damage, liver fibrosis, and hepatocellular carcinoma (HCC) in rare instances [3]. In general, an unbalanced diet and a lack of physical activity are the main causes of NAFLD [4]. NAFLD is considered a metabolic syndrome (MetS) issue because of the accumulating evidence that it is associated with obesity [5,6,7,8], insulin resistance [9,10,11,12], type 2 diabetes [9,13,14,15,16], and dyslipidaemia [17,18]. The main causes of the onset and development of NAFLD in MetS include lipotoxicity, inflammation, and oxidative stress [19]. Currently, NAFLD lacks efficacious targeted pharmacological interventions, and therefore, treatment strategies primarily involve modifying one’s dietary and lifestyle habits, protecting liver function, reducing cholesterol levels, and enhancing insulin sensitivity [20,21].

Natural products provide a diverse spectrum of new and unique bioactive compounds for drug discovery and offer a refreshed strategy [22]. Due to its relative convenience, safety, and efficacy, the utilisation of natural medicine is on the rise worldwide [23]. Carotenoids, a naturally occurring class of isoprenoid pigments, have been proven to inhibit NAFLD growth by exerting antioxidant, lipid-lowering, anti-inflammatory, anti-fibrotic, and insulin-sensitising effects. Researchers investigated the prevention of the onset and progression of NAFLD by carotenoids like astaxanthin, lycopene, β-carotene, β-cryptoxanthin, lutein, fucoxanthin, and crocetin [24]. Astaxanthin is a xanthophyll carotenoid with a dark red colour and a chemical makeup of 3,3′-dihydroxy-4,4′-diketo-β,β’-carotene that is present in several microorganisms and marine creatures, including the green microalgae *Haematococcus pluvialis*, salmon, prawns, crabs, etc. [25]. *H. pluvialis*, a freshwater microalga in the Chlamydomonadaceae family, is well known as a major source of naturally occurring astaxanthin [26]. The chemical structure of astaxanthin facilitates its cellular permeability and ability to counteract active oxygen and free radicals. Hence, it is recognised for its robust antioxidant properties, surpassing those of vitamin E by 500 times higher [27]. As an antioxidant, astaxanthin exhibits 10 ten times greater activity than other carotenoids (such as lutein, β-carotene, and zeaxanthin) and 100 times the activity of α-tocopherol [28]. Studies have shown that astaxanthin significantly reduces the risk of developing ischemia, NAFLD, liver cancer, and liver fibrosis. Its mechanism is linked to antioxidant and anti-inflammatory properties besides the control of many signalling pathways. Astaxanthin is one of the most crucial components in treating NAFLD and is regarded as the best antioxidant among carotenoids [29]. Astaxanthin may help avoid the onset of NAFLD in many ways. 

The objective of this study is to conduct a comprehensive analysis of the existing literature on the function of astaxanthin in the context of NAFLD. The search keyword “(NAFLD OR fatty liver) AND Astaxanthin” returned 66 results in PubMed. The results obtained 48 research papers, 16 review papers, and 2 letters to the editor. The time range based on ‘all years’ resulted in the paper being published between 1993 and 21 February 2023. In this review, 12 research articles and 1 letter to the editor were selected for this study. The selection criteria for the article were based on the subject of the study.

## 2. Astaxanthin’s Effect and Underlying Mechanisms in NAFLD

### 2.1. Clinical Studies

Table 1 summarises the human studies on the effects of astaxanthin on NAFLD. Nie et al. [30] presented a clinical trial involving 12 patients with biopsy-confirmed NASH who were administered astaxanthin or placebo orally for 24 weeks. After 24 weeks of astaxanthin supplementation, plasma parameters associated with glucose and lipid metabolism and liver functions remained unchanged. No statistically significant disparity was observed between the patients in the placebo group and those who were administered astaxanthin concerning changes from their respective baselines. However, astaxanthin therapy substantially reduced hepatic steatosis in patients with NASH. Furthermore, the administration of placebo therapy did not significantly impact the NAFLD activity score (NAS). Conversely, astaxanthin therapy resulted in reduced steatosis grade and improved in lobular inflammation, albeit any discernible effects on the presence of ballooning or the level of fibrosis. The findings indicate that astaxanthin can reduce the overall NAS score and improve NASH in human subjects [30]. Astaxanthin shows the ability to delay the development of NASH in humans. As indicated by preliminary research findings, it can act as a new and promising treatment option for NASH.

Studies revealed that 50% of individuals affected by Werner syndrome (WS) exhibit complex fatty liver disease and a substantial incidence of NASH. A case was reported in which astaxanthin improved NAFLD in a 58-year-old lady with WS and diabetes mellitus [31]. Astaxanthin has been shown to ameliorate NAFLD in WS patients with diabetes mellitus. No side effects were noted after starting 12 mg of astaxanthin per day. The results of computed tomography (CT) scans conducted three and six months after the initiation of astaxanthin intake revealed a significant improvement in the severity of the fatty liver. The mechanism by which astaxanthin improved NAFLD in the female subject remains uncertain, as there were no observed changes in oxidative stress or inflammation markers subsequent to astaxanthin administration. Hence, to assess the efficacy of astaxanthin in the future, population-based, randomised controlled trials are required. Astaxanthin may therefore become the main therapy option in the future for patients with fatty liver disease, particularly those with WS [31]. It is important to note that there are yet not enough human data to determine how astaxanthin medication affects NAFLD in actual clinical settings. It is required to conduct more clinical research on the significance of astaxanthin for liver protection.

### 2.2. In Vivo Studies

In vivo studies investigating the effect of astaxanthin on NAFLD are summarised in Table 2. The latest study conducted by Nguyen-Lee et al. [29] evaluated astaxanthin’s potential to lower blood lipids and liver fat in vivo. The present study involved administering astaxanthin to mice fed a high-fat diet (HFD) to assess the efficacy of the treatment in preventing NAFLD in the mouse model. The study was split into two stages. During the initial stage, which spanned 16 weeks, the mice were assigned to three groups: normal diet group, HFD group, or astaxanthin group. The second phase was extended for an additional 8 weeks, after which the astaxanthin administration was stopped while the same diet was continued. The findings demonstrated that astaxanthin was not only a highly effective substance for preventing liver lipid accumulation and high blood cholesterol, but it also kept the glucose levels stable, which was beneficial for preventing metabolic illnesses brought on by food. The aspartate aminotransferase (AST) levels were significantly lower in the astaxanthin group than in the HFD mice. The administration of the astaxanthin supplement demonstrated the capacity to preserve typical hepatic function in contrast to the HFD group. After eight weeks following the discontinuation of astaxanthin, the blood AST levels in the control and astaxanthin groups were observed to have increased. This suggests that the hepatosteatosis-reducing function of astaxanthin had ceased, leaving the liver unprotected. The Swiss mouse model exhibited weight gain upon the administration of astaxanthin; nonetheless, it demonstrated efficacy in modulating of biochemical parameters. Astaxanthin exhibited the potential as a compound for hepatosteatosis prevention and hyperlipidaemia in mice fed an HFD. However, it did not demonstrate efficacy in weight control. The effects of astaxanthin were observed to decrease during the extended period of astaxanthin withdrawal, necessitating further investigation for future applications. The authors proposed that forthcoming studies should encompass an assessment of diabetes and the utilisation of a new natural substance in conjunction with astaxanthin to sustain proportional weight increases in models of obesity induced by dietary factors [29]. 

Wang and colleagues [32] investigated the role of astaxanthin in regulating liver lipid metabolism and gut microbiota. The investigation involved the oral administration of astaxanthin at concentrations of 0.25%, 0.5%, and 0.75% in conjunction with an HFD to mice for nine weeks. Besides improving hepatic steatosis and oxidative stress, the study indicated that astaxanthin could potentially reduce body weight gain, lipid droplet formation, and hepatic triglycerides. The administration of astaxanthin resulted in a significant reduction of total cholesterol (TC), triglycerides (TG), and low-density lipoprotein cholesterol (LDL-C) levels in mice. The group administered with 0.75% astaxanthin exhibited a significant decrease in the expression levels of acetyl-CoA carboxylase (ACC), Fas cell surface death receptor (FAS), and stearoyl-CoA desaturase 1 (SCD-1) compared to the HFD group. Conversely, the expression of genes associated with lipid oxidation and bile acid metabolism, i.e., carnitine palmitoyltransferase-1 (CPT-1), liver X receptor alpha (Lxrα), cholesterol 7α-hydroxylase1 (CYP7A1), and mitochondrial enzyme sterol 27-hydroxylase (CYP27A1), was found to be elevated in the group treated with 0.75% astaxanthin. These findings showed that an HFD increased fat synthesis, ultimately disrupting lipid metabolism. In addition, high doses of astaxanthin may be able to treat this disorder by increasing cholesterol metabolism and decreasing fat synthesis. At a concentration of 0.75%, astaxanthin altered the levels of 34 lipid metabolites relevant to hepatic cholesterol and fatty acid metabolism. This alteration may have been caused by the upregulation of genes related to bile acid production and the repression of genes related to lipogenesis. By preventing the formation of obesity-related *Parabacteroides* and *Desulfovibrio* while encouraging the growth of *Allobaculum* and *Akkermansia*, astaxanthin reduces the dysbiosis of the gut microbiota caused by HFD. According to the research, dietary astaxanthin may lower the incidence of metabolic disease by modifying the liver–gut axis. Finally, this research provides evidence for the effectiveness of astaxanthin in preventing obesity and the functional effects that go along with it [32].

Yang et al. [33] examined the effects of astaxanthin on the advancement of NASH in mice subjected to a choline-deficient, L-amino acid-defined, and high-fat diet (CDAHFD). Their findings suggest that oral astaxanthin administration may positively impact NASH and liver fibrosis through the regulating hepatic immune response, inflammation, and oxidative stress. The introduction of astaxanthin led to a decline in the infiltration of macrophages derived from monocytes in the liver. Additionally, it caused a decrease in the activation of hepatic stellate cells, the reaction to oxidative stress, and the mortality of hepatocytes. Likewise, a reduction in the hepatic expression of proinflammatory cytokines, such as tumour necrosis factor-alpha (TNF-α), transforming growth factor-beta 1 (TGF-β1), and interleukin-1β (IL-1β), was observed concomitant with this occurrence. A proteomic study was conducted to examine the effect of astaxanthin on the suppression of oxidative stress. The administration of astaxanthin resulted in decreased in the oxidative stress response observed in the livers of mice fed with HFD. This effect was attributed to the downregulation of various proteins, matrix metallopeptidase 9 (Mmp9), solute carrier family 25-member 24 (Slc25a24), glucose-6-phosphate dehydrogenase X-linked (G6pdx), annexin A1 (Anxa1), dynamin 2 (Dnm2), cofilin 1 (Cfl1), superoxide dismutase 3 (SOD3), proliferating cell nuclear antigen (Pcna), and poly (ADP-ribose) polymerase family, member 1 (Parp1). The findings indicate that astaxanthin exhibits anti-oxidative stress properties in the development of NASH induced by an HFD. Overall, the findings suggest that astaxanthin could be a feasible therapeutic alternative for individuals experiencing NASH and hepatic fibrosis. Similarly, Wu et al. [27] studied mice were given either a high-fat or chow diet for up to 12 weeks, with or without astaxanthin. Astaxanthin altered lipid metabolism in the livers of NAFLD mice and avoided fibrosis and inflammation. In mice given an HFD, astaxanthin decreased liver damage by regulating lipid metabolism, fibrosis, and inflammation. To exert its anti-NAFLD effects, astaxanthin triggered the FGF21/PGC1 pathway. CYP3A and OATP1A/1B help remove larotrectinib from part of the body. They concluded that astaxanthin shows promise as a medicine for alleviating or treating NAFLD [27]. 

The impact of astaxanthin on acute hepatic ischemia-reperfusion injury (IRI) in a steatotic mouse liver model fed with a methionine- and choline-deficient high fat (MCDHF) diet was explored by Li et al. [34]. The administration of astaxanthin through IRI resulted in a significant reduction in the levels of alanine aminotransferase (ALT) and AST in the bloodstream besides a decrease in the quantity of terminal deoxynucleotidyl transferase dUTP nick end labelling (TUNEL), F4/80, or 4HNE-positive cells and downregulation of the mRNA levels of cytokines that are typically associated with inflammation in mice with MCDHF diet-induced liver injury. Astaxanthin decreased the inflammatory cytokine expression but enhanced the expression of heme oxygenase-1 (HO-1), hypoxia-inducible factor 1-alpha (HIF-1α), the phosphorylation of Akt, and a mammalian target of rapamycin (mTOR) under IR injury. This suggests that astaxanthin’s preconditioning effects are mediated via the HIF pathway, resulting in increased protection. According to Li et al. [34], administering astaxanthin as a pre-treatment for IRI, particularly in liver transplantation involving a steatotic liver, has been found to have a dependable therapeutic effect and protection.

Kim et al. [35] investigated the potential of astaxanthin in reducing the metabolic imbalances, inflammation, and fibrosis connected to obesity in diet-induced obesity (DIO) and NASH animal models. Compared to the low-fat diet (LF), astaxanthin significantly decreased plasma TC, TG, and glucose in the high-fat/high-sucrose (HF/HS) group. Astaxanthin decreased hepatic mRNA levels of macrophage and fibrosis markers in both models. The astaxanthin impact was more prominent in NASH animals than in DIO mice. After lipopolysaccharide (LPS) treatment, astaxanthin markedly decreased the quantity of TNF mRNA in the splenocytes of DIO mice compared to the control animals fed an HF/HS diet. Furthermore, astaxanthin significantly increased the levels of genes that regulate fatty acid β-oxidation and mitochondrial biogenesis in skeletal muscle compared to the control obese mice but did not affect the levels of adipose lipogenic genes. By lowering inflammation and fibrosis in the liver and adipose tissue, astaxanthin increases the capacity of skeletal muscle to burn fat in the mitochondria in obese mice. However, additional research is required to determine how astaxanthin improves skeletal muscle energy utilisation while reducing M1 macrophage infiltration in liver and adipose tissue fibrosis [35]. 

Xu et al. [36] examined the impact of a blend of flaxseed oil (FO) and astaxanthin on hepatic lipid accumulation and oxidative stress in rats that were administered an HFD. The replacement of lard with FO containing astaxanthin has been found to decrease fat accumulation in the liver (steatosis) and lower hepatic TG and TC levels. The co-administration of FO and astaxanthin resulted in a notable reduction in hepatic sterol regulatory element-binding transcription factor 1 (SREBP-1c) and 3-hydroxy-3-methylglutaryl-CoA reductase (HMGCR) while simultaneously increasing the expression of peroxisome proliferator-activated receptor (PPAR). The administration of FO and astaxanthin resulted in a significant reduction in the expression of fatty acid synthase (FASN) and acetyl CoA carboxylase (ACC) while simultaneously inducing the expression of CPT-1 and acyl CoA oxidase (ACO). Contrarily, the administration of FO and astaxanthin resulted in a significant increase in the activity of hepatic SOD, catalase (CAT), and glutathione peroxidase (GPx) and an increase in GSH levels. Additionally, a notable decrease in hepatic lipid peroxidation was noted. FO and astaxanthin administration might facilitate the capacity to mitigate NAFLD through the reduction of lipis synthesis, oxidative stress, and the reversal of hepatic steatosis. Although the potential of FO and astaxanthin for hepatoprotection has been suggested, further research in NAFLD models is required to validate this assertion, as stated by Xu et al. [36].

Kobori and colleagues [37] examined the hepatic gene expression patterns in mice with NASH induced by a high-cholesterol, high-cholate, and high-fat (CL) diet following the administration of astaxanthin or vitamin E. The study revealed that both astaxanthin and vitamin E enhanced gene expression related to eukaryotic initiation factor-2 (EIF2) signalling. This signalling pathway is known to be suppressed in NASH due to the endoplasmic reticulum (ER) stress in the liver. However, the administration of astaxanthin did not yield a statistically significant augmentation in the transcriptional activity of genes linked to mitochondrial dysfunction. The hypothesis put forth by the authors suggests that astaxanthin could inhibit the functions of ligand-dependent nuclear receptors, specifically the peroxisome proliferator-activated receptor alpha (PPARα) and peroxisome proliferator-activated receptor delta (PPARδ) as well as their associated constituents. Therefore, to establish an astaxanthin-based treatment regimen for individuals with NASH, it is imperative to gain a comprehensive understanding of the functioning of PPARα and associated molecules in the livers of mice afflicted with NASH that was induced by dietary factors, as per the findings of [37].

Ni et al. [30] also compared the effects of astaxanthin and vitamin E on NASH. In mice given an HFD, astaxanthin decreased the excessive liver lipid build-up and peroxidation in a lipotoxic model of NASH. M1-type macrophages/Kupffer cells and activated stellate cells were increased while hepatic fibrosis and inflammation were reduced. Insulin resistance and hepatic inflammation were improved by astaxanthin’s M2-dominant shift in macrophages/Kupffer cells, whereas CD4+ and CD8+ T cell recruitment in the liver was reduced. Reversing insulin resistance, hepatic inflammation, and fibrosis in patients with pre-existing NASH is crucial. Despite the crucial roles that oxidative stress and insulin resistance play in the development of NASH, the cure for the condition has been documented. The lipogenesis gene, including Srebp1c, Lxrα, carbohydrate-responsive element-binding protein (Chrebp), and the fatty acid synthesis genes, i.e., FASN and Scd1, were inhibited by astaxanthin. The consumption of a cholate diet increased the expression of platelet glycoprotein 4 (CD36), which was subsequently reduced by astaxanthin, while vitamin E had no significant effect on its expression. The findings indicate that astaxanthin inhibited lipogenesis and lipid uptake, thereby mitigating lipid accumulation in the liver of mice with NAFLD/NASH. Overall, astaxanthin beats vitamin E in treating and preventing NASH in mice [30].

Nguyen-Le et al. [38] conducted a study using the Swiss mouse model to investigate the impact of astaxanthin on the body weight and liver weight of mice that were fed with an HFD. During phase 1 and phase 2 of the study, mice fed with an HFD were administered astaxanthin orally for 16 weeks and 8 weeks. Following a 16 week period of treatment with astaxanthin, a reduction in both body weight and adipose tissue was observed in the mice. The removal of astaxanthin for 8 weeks after the completion of phase 1 resulted in a significant rise in the liver-to-body weight ratio after 24 weeks. It is possible that stopping astaxanthin administration may have resulted in the loss of its effect. The efficacy of astaxanthin in mitigating hepatosteatosis in mice fed with an HFD was demonstrated by its ability to down-regulate peroxisome proliferator-activated receptor gamma (PPARγ) and stabilise PPARα. The properties of astaxanthin demonstrate the efficient regulation of liver lipid homeostasis. Nguyen-Le et al. [38] suggested that astaxanthin may be a promising compound in preventing hepatitis steatosis.

Yang et al. [39] employed male C57BL/6J mice as subjects to examine the potential of astaxanthin supplementation to alleviate the onset of NAFLD in DIO mice. Over 12 weeks, the mice were administered an HFD enriched with astaxanthin at varying concentrations of 0%, 0.003%, 0.01%, or 0.03%. The administration of astaxanthin at a concentration of 0.03% reduced plasma levels of triacylglycerol (TAG), ALT, and AST. Additionally, it upregulated the expression of endogenous antioxidant genes in the liver and decreased the susceptibility of splenocytes to activation by LPS. These findings suggest that astaxanthin supplementation at a concentration of 0.03% exerts a hypotriacylglycerolaemic effect. The hepatic expression of nuclear factor erythroid 2–related factor 2 (Nrf2) and its downstream genes, i.e., SOD1, glutamate–cysteine ligase regulatory subunit (GCLm), and glutathione peroxidase 1 (GPx-1), exhibited a significant increase in the group administered with 0.03% astaxanthin. According to Yang et al. [39], astaxanthin can mitigate inflammation and metabolic irregularities associated with obesity.

One potential approach for safeguarding the liver is using natural supplements. It is noteworthy that previous studies on animals showed the impact of astaxanthin intervention on NAFLD. The protective and therapeutic potential of astaxanthin on NAFLD is evident from the findings of 11 animal model studies using different type of animal models, as described above. It is imperative to consider that hepatic pathologies may remain inconspicuous and devoid of symptoms in apparently healthy individuals. Thus, it is essential to develop strategies for safeguarding the health of the liver.

### 2.3. In Vitro Studies

Table 3 presents a summary of the impact of astaxanthin on NAFLD as observed in in vitro models. Wu et al. [27] examined the impact of astaxanthin on L02-induced steatosis cells by utilising human L02 cells. The cells were exposed to various amounts of astaxanthin and free fatty acids (FFAs) in combinations over 48 h. Astaxanthin was found to increase cellular survival rates by approximately 18%. The expression of Bax and caspase 9 was inhibited by astaxanthin. The results indicate that astaxanthin significantly reduced in lipid concentration in L02 hepatocytes exposed to FFA. The administration of astaxanthin resulted in modifications in the gene expression patterns related to lipid metabolism. Specifically, the expressions of Apolipoprotein B (ApoB), Apolipoprotein E (ApoE), CPT-1, fibroblast growth factor 21 (FGF21), forkhead box-containing transcription factors (FOXA), PGC-1α, PPAR-α, transmembrane 6 superfamily member 2 (Tm6sf2), and uncoupling protein 1 (UCP1) were upregulated while the expressions of CD36, fatty acid transport protein-5 (FATP5), adipose differentiation-related protein (ADRP), and SREBP-1c were downregulated. The results of the study indicated that astaxanthin protected L02 hepatocytes against damage induced by FFA. Furthermore, the administration of astaxanthin demonstrated a favourable impact on the modulation of mitochondrial function. The exposure to astaxanthin resulted in reduced of reactive oxygen species (ROS), an increase in the quantity of mitochondrial DNA (mtDNA) copies, an elevation in the mitochondrial membrane potential (ΔΨm), and an upregulation in the expression of nuclear respiratory factor 1 (Nrf1) and mitochondrial transcription factor A (TFAM) in hepatocytes.

Li et al. [34] looked into the anti-inflammatory activities of astaxanthin in Kupffer cells and its anti-apoptotic effects in hepatocytes treated with hypoxia and reoxygenation (HR). The amount of ROS in Kupffer cells isolated from steatotic livers subjected to hepatic reperfusion significantly decreased after astaxanthin treatment. The mRNA expression of inflammatory cytokines, specifically IL-1β and TNF-α, produced by Kupffer cells and treated with HR, was inhibited by astaxanthin. In contrast to the control group, astaxanthin increased the expression of HO-1 and Nrf2 and activated the Akt/mTOR/HIF-1α pathway in Kupffer cells obtained from steatotic livers. On the contrary, astaxanthin reduced apoptosis by the adjusting of the Bax/Bcl-2 ratio and caused a drop in the phosphorylated levels of p38, extracellular signal-regulated kinase (ERK), and c-Jun NH2-terminal kinase (JNK) in steatotic hepatocytes extracted from steatotic livers subjected to HR.

Nie et al. [30] examined the effect of astaxanthin on lipid accumulation in vitro using primary hepatocytes. These hepatocytes were incubated with astaxanthin or α-tocopherol. The findings showed that incubation with astaxanthin reduced TG formation in lipid-loaded primary hepatocytes in a dose-dependent way, as shown by Oil Red O staining and the cellular TG level. However, no such impact was noticed in α-tocopherol. According to the findings, astaxanthin may lessen the build-up of fat in hepatocytes. Astaxanthin treatment did not affect the mRNA levels of the lipogenic and fatty acid oxidation genes in between treatments. However, oleic acid treatment significantly increased the expression of CD36, a crucial regulator of lipid uptake; in contrast, astaxanthin significantly decreased CD36 expression in a dose-dependent manner. Furthermore, astaxanthin did not have an effect on the phosphorylation levels of p38 MAPK and c-Jun in hepatocytes exposed to oleic acid. Overall, the results show that astaxanthin improved the simple fatty liver by lowering the lipid uptake, which in turn reduced lipid build-up [30]. 

Yang et al. [40] investigated the role of astaxanthin in regulating the expression of fibrogenic genes in human hepatic stellate cells (HSCs) and primary mouse HSCs. Astaxanthin reduced the generation of tert-butyl hydrogen peroxide-induced ROS generation and transforming growth factor 1 (TGF-1) in LX-2 cells and significantly decreased the amounts of procollagen type 1, alpha 1 (Col1A1), α-smooth muscle actin (α--SMA) mRNA, and α-SMA protein. Additionally, it prevented SMAD family member 3 (Smad3) from becoming phosphorylated and moving into the nucleus. Astaxanthin suppressed the activation of the Smad3 pathway in HSCs and had anti-fibrogenic effects via blocking TGF-1 signalling. This study suggests that astaxanthin may be utilised to cure or prevent hepatic fibrosis [40]. 

The gatekeepers of liver homeostasis in healthy settings are liver sinusoidal endothelial cells (LSECs). An essential structural feature of healthy LSECs is the presence of fenestrae, which are open transmembrane pores. The hepatic endothelial fenestrae are highly dynamic structures that function as a selective barrier, regulating the extensive exchange of substances between the bloodstream and the liver parenchyma. Changes in the quantity or size of fenestrae induced by hormones, drugs, toxins, or pathological conditions can give rise to significant disruptions in hepatic functionality [41]. Meanwhile, NAFLD are impacted by a “multiple parallel-hit model” wherein oxidative stress assumes a central role. LSEC defenestration may represent a significant pathogenic mechanism influencing the development of NAFLD. The antioxidants’ ability to lower ROS and Rho/ROCK (Rho-associated protein kinase) signalling would encourage the formation of fenestration [42]. Astaxanthin noticeably decreased oxidative stress and ROS in Kupffer cells [34] or HSCs [40], suggesting that it may also be able to do the same in LSECs. For instance, it may help with the formation and functioning of the hepatic fenestrae. Despite the abundance of evidence connecting oxidative stress to liver disease, not much is known about how “antioxidant” compounds could affect the LSEC phenotype, necessitating further research to address this issue.

## 3. Limitations and Future Perspectives

Although astaxanthin has some promising pharmacological properties, there is yet inadequate human data to investigate the effect of astaxanthin therapy on liver function in clinical settings. A limited clinical trial is insufficient to assert that astaxanthin helps liver disease. More information from human trials is required before confirming astaxanthin’s liver-protective properties. Although astaxanthin has been well-examined for tissue distribution, absorption, and toxicity in experimental animals, there is a dearth of information from clinical trials. Therefore, extensive epidemiological and clinical studies are required to prove its continued use [43]. Larger-scale clinical trials are required to corroborate these findings. Future human clinical studies need to be planned for the use of astaxanthin as a dietary supplement or nutraceutical. Further research into the role of astaxanthin in liver protection is required to better explore the possibility of astaxanthin’s preventive and therapeutic effects against liver diseases in human trials. 

Humans cannot produce astaxanthin; hence, it must be taken as a supplement. This necessitates the development of a suitable astaxanthin nano-formulation in the near future to improve its dissolution, bioavailability, and therapeutic applications, particularly for the liver. However, the safety profile of astaxanthin is uncertain due to the scarcity of well-designed clinical research. Astaxanthin is considered safe when consumed in food doses and possibly safe when taken as a supplement. According to Lorenz and Cysewski [44], the United States Food and Drug Administration (USFDA) recognised *H. pluvialis*-derived astaxanthin as Generally Recognized as Safe (GRAS) in 1999 and can be used as a nutraceutical ingredient for ingestion. In addition, the European Food Safety Authority (EFSA) has authorised the use of astaxanthin in dietary supplements as nutraceuticals with a daily intake limit for humans of 0.034 mg/kg [45]. A published patent of ‘Methods for Reversing Fibrosis’ by Ji-Young [46] offers strategies for treating fibrosis and fibrotic disorders by inhibiting the activation of hepatic stellate cells using astaxanthin. Other patents emphasise astaxanthin as a novel, safe, and highly effective agent for preventing insulin resistance and metabolic syndrome [47]. The current invention relates to a pharmaceutical or food composition used to treat or prevent alcoholic liver disease that contains astaxanthin as an active ingredient. Astaxanthin can treat, prevent, and alleviate alcoholic liver disease [48]. Nonetheless, to our knowledge, astaxanthin’s specific impact on NAFLD has not yet been the subject of a patent report.

Astaxanthin has been proven to have limited oral bioavailability since it is a highly lipid-soluble carotenoid, restricting its therapeutic applicability. Studies indicate that the oral bioavailability of astaxanthin is comparatively limited, typically falling within the range of 10–50% for a given dosage [49,50,51,52]. This is attributed to its inadequate solubility in water and lipid-based blood constituents, including triglycerides. Astaxanthin monoester and diester were synthesised by Fukami et al. [53] to administer them in a rat model. The findings indicate that the bioavailability of astaxanthin monoester was better than that of the diester. Moreover, the hepatic maximum metabolic concentration of astaxanthin monoester was observed to be thrice as high as that in serum, indicating a significant correlation between astaxanthin’s fatty acid chain composition and its bioavailability. However, additional clarification is necessary to fully comprehend this association [53]. In recent times, there has been a development of novel nano-formulations aimed at enhancing their bioavailability, and several suggestions have been put forward. The utilisation of delivery systems for astaxanthin, including nano emulsions, liposomes, solid lipid nanoparticles, and chitosan- and PLGA-based nanoparticles, has exhibited enhanced oral bioavailability of astaxanthin [23]. The chemical structure of astaxanthin can be further enhanced to create more durable and stable formulations that can be incorporated into food and pharmaceutical products to promote human health, particularly in the context of NAFLD.

## 4. Conclusions

**Figure 1:**
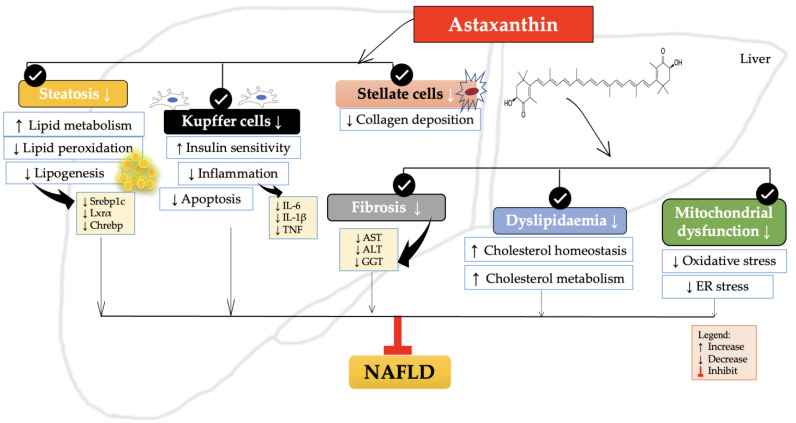
The schematic effects of astaxanthin on the molecular targets associated with NAFLD. The therapeutic efficacy of astaxanthin in reducing fibrosis, steatosis, inflammation, dyslipidaemia, and other related conditions associated with NAFLD has been suggested by its potential effects. Legends for figure—ALT: alanine aminotransferase; AST: aspartate aminotransferase; Chrebp: carbohydrate-responsive element-binding protein; ER: Endoplasmic reticulum; GGT: gamma-glutamyl transferase; IL-1β: Interleukin-1β; IL-6: Interleukin 6; Lxrα: liver X receptor alpha; Srebp1c: sterol regulatory element-binding protein 1c; TG: triglycerides; TNF: tumor necrosis factor.

The current study provides a summary of the function of astaxanthin in NAFLD and its related mechanisms. Astaxanthin is widely recognised for its potent antioxidant properties. It has been demonstrated to regulate diverse molecular and cellular processes in liver disease through clinical trials, in vivo, and in vitro cell experiments. Research has demonstrated that astaxanthin possesses preventive and therapeutic properties in NAFLD through diverse mechanisms, as illustrated in Figure 1. The significant impact of astaxanthin on NAFLD indicates its potential as a viable therapeutic alternative for the treatment of fibrosis, steatosis, inflammation, dyslipidaemia, and other related conditions. Astaxanthin exhibits promising potential as a viable pharmaceutical agent for preventing or treating NAFLD in the foreseeable future.

## Figures and Tables

**Table 1:** Human trials examining the effect of astaxanthin on NAFLD.

References	Subjects and Experiment Duration	Experimental Groupings	Effects
[30]	12 biopsy-confirmed NASH patients 24 weeks treatment	Placebo and astaxanthin 12 mg/day	↓ Steatohepatitis ↓ Grade of steatosis ↓ NAS ↓ Progression of NASH
[31]	58-year-old woman with Werner syndromeand diabetes mellitus 3 and 6 months	Treatment with astaxanthin 12 mg/day	↑ Liver-to-spleen Hounsfield units ↓ Severity of fatty liver ↓ AST ↓ ALT ↓ GGT

↑: up-regulation; ↓: down-regulation; AST: aspartate aminotransferase; ALT: alanine aminotransferase; GGT: gamma-glutamyl transferase; NAS: NAFLD activity score.

**Table 2:** Effects of astaxanthin on NAFLD in animal studies.

References	Animal Model and Duration of Experiment	Experimental Groupings	Effects	Mechanisms
[29]	Female Swiss mice 16 weeks and 8 weeks	3 groups: Normal diet; HFD; HFD + astaxanthin (30 mg/kg BW/day First phase: Normal diet, HFD + astaxanthin Second phase (prolonged for 8 weeks): Normal diet, HFD + astaxanthin (astaxanthin supplementation was discontinued) group	↑ BW ↓ Hepatosteatosis ↓ Lipid droplets ratio ↓ AST ↓ TC ↓ TG ↓ LDL-c Maintaining blood cholesterol	**-**
[32]	Male C57BL/6 mice 9 weeks	5 groups: Normal diet; HFD; HFD + 0.25% astaxanthin; HFD + 0.5% astaxanthin; HFD + 0.75% astaxanthin	↓ BW gain ↓ Energy intake ↓ Liver wet weights ↓ TC, ↓ TG ↓ LDL-C, ↓ AST ↓ Lipid peroxidation ↓ Steatohepatitis scores ↓ Fatty droplets ↓ Apoptosis ↑ Lipid metabolism ↑ Cholesterol metabolism ↓ Fat synthesis	↑: CAT, SOD, GSH, CPT-1,Lxrα, CYP7A1, CYP27A1 ↓: ACC, FAS, SCD-1
[33]	Male C57BL/6 mice 6 weeks	4 groups: Normal diet; CDAHFD; HFD; HFD + astaxanthin (80 mg/kg of mouse BW)	↓ Liver-to-BW ratio ↓ Infiltration of inflammatory cells ↓ Hepatocyte death ↓ aHSCs ↓ Infiltration of monocyte-derived macrophages ↓ Oxidative stress	↓: Collagen α-SMA, Col1α1, Col4α1, IL-1β, TGF-β1, TNF-α, bFGF, Slc25a24, Mmp9, Anxa1, G6pdx, SOD3, Pcna, Parp1, Dnm2, Cfl1
[27]	Male C57 10 weeks	6 groups: Control: standard chow diet; HFD: HFD + saline every 2 days in later 10 weeks; HFD + astaxanthin: 10, 30, and 60 mg/kg of astaxanthin by gavage every 2 days in later 10 weeks; HFD + tail-vein injection of control-siRNA 8x (first 4 weeks); HFD + tail-vein injection of FGF21-siRNA 8x during (first 4 weeks); HFD + tail-vein injection of FGF21-siRNA 8x (first 4 weeks) + 60 mg/kg of astaxanthin by gavage every 2 days during the later 10 weeks	↓ BW, ↓ NAS ↓ Liver weight ↓ Serum TG ↓ AST, ↓ ALT ↓ Steatosis ↓ Lobular inflammation ↓ Hepatocellular ballooning ↓ Hepatic lipid deposition ↓ Enlargement of adipocytes ↓ Fatty acid uptake and synthesis ↑ Lipid decomposition ↑ Fatty acid oxidation ↓ Hepatic inflammation	↑: ApoB, ApoE, CPT1α, FGF21, FOXA, PGC-1α, PPARα, Tm6 ↓: Bax, Caspase 9. TNF-α, IL-1β, iNOS, Collagen I, TGF-β1, α-SMA, CTGF, ADRP, CD36, FATP5, SREBP-1c
[34]	Male C57BL/6 mice 3 weeks	3 groups: MCDHF group: olive oil by gavagewith shame operation MCDHF IR group: olive oil by gavage before IR MCDHF IR + ASTX group: astaxanthin (25 mg/kg) for 48, 24 h and 40 min	↓ TUNEL-positive cells F4/80-positive cells ↓ 4-HNE-positive cells ↓ AST ↓ ALT	↑: HO-1, HIF-1α ↓: IL-6, OPN, iNOS
[35]	Male C57BL/6J mice 30 weeks (DIO model) and 18 weeks (NASH model)	5 groups: DIO model: Low fat (6% fat, *w/w*); High fat/high sucrose (HF/HS): 35% fat, 35% sucrose (*w/w*); HF/HS + astaxanthin (AHF/HS): 0.03% astaxanthin (*w/w*) NASH model: HF/HS + 2% cholesterol (HF/HS/HC); HF/HS/HC + 0.015% astaxanthin	↓ TC ↓ TG ↓ Glucose ↓ Inflammation ↓ Fibrosis	DIO model: ↓: F4/80, CD11c, MCP-1, Caspase 3, Collagen type I & VI, COL1A1, COL6A1, COL6A3, LOXL2, HIF-1α, FAS, SCD-1, LUM; ↑: Arg-1, CPT-1α, Acox-1, PPARα, PGC-1α NASH model: ↑: CD206; ↓: F4/80, CD68, CD11c, MCP-1, Lumican, Vimentin, COL1A1, COL6A1, COL6A3, MMP2, HIF-1α, TGFβ1, TNC
[36]	Male Sprague–Dawley rats 10 weeks	4 groups: HFD; L-FO + astaxanthin group: 75% lard + 25% flaxseed oil (FO) + astaxanthin M-FO + astaxanthin group: 50% lard + 50% FO + astaxanthin H-FO + astaxanthin group: 100% FO + astaxanthin	↓ Circular lipid droplets ↓ Steatosis ↓ Hepatic TG	↑: PPARα, CPT-1, ACO, SOD, CAT, GPx, GSH ↓: SREBP1, HMGCR, FAS, ACC, TBARs
[37]	C57BL/6J male mice 12 weeks	4 groups: Normal diet; CL diet: 60% calories from fat, 1.25% cholesterol, 0.5% sodium cholate); CL diet + 0.02% astaxanthin; CL diet + 0.02% vitamin E	↑ EIF2 ↑ mTOR signalling	↑: Akt2, RICTOR, ERN1,PML, MAP3K8, IKZF1, CD28 ↓: Cpt1a, Acox1, Scd1, PNPLA2, PPARD, PPARα,RXRA, IL-6
[30]	Male C57BL/6J mice and male ob/ob mice 10 weeks (hepatic model) 12 weeks (NASH model)	9 groups: Hepatic model: Normal chow (NC): 10% of calories from fat; HFD; HFD + 0.02% astaxanthin NASH model: NC; NC + 0.02% astaxanthin; NC + 0.02% vitamin E; High-fat, cholesterol, and cholate diet (CL diet): 60% of calories from fat, 1.25% cholesterol, 0.5% sodium cholate); CL + 0.02% astaxanthin; CL + 0.02% vitamin E	Hepatic model: ↓ Steatosis ↓ TG NASH model: ↓ Dyslipidaemia ↓ Lipid peroxidation ↓ Blood glucose ↑ Insulin sensitivity ↓ F4/80+ cells ↓ Kupffer cells ↓ Hepatic inflammation ↓ Total macrophage ↓ TG, ↓ TC, ↓ NEFA ↓ AST, ↓ ALT	**↓**: Srebp1c, Lxrα, Chrebp, FASN, Scd1, CD36, p38, MAPK, NF-κB p65, ERK, F4/80, Tnf, IL-6, IL-1β, α-SMA, TGF-β1, Col1a1, PAI-1, Cd11c, iNOS, MCP-1, Ccr2 ↑: p-IRβ/IRβ, p-Akt/Akt, CD163, CD206, Il10, Chi3l3, Mgl1
[38]	Female Swiss Mice 16 weeks and 8 weeks	3 groups: Phase 1 (16 weeks): Normal diet; HFD: 60% total calories from fat; HFD + astaxanthin (30 mg/kg BW) Phase 2 (8 weeks): Normal, HFD and HFD + astaxanthin (astaxanthin was terminated after 16 weeks)	↓ Fat/BW ↓ Liver weight	↓: PPARγ, PPARα
[39]	Male C57BL/6J mice 12 weeks	4 groups: HFD control diet (35%, *w/w*); HFD + 0.003% of astaxanthin; HFD + 0.01% of astaxanthin; HFD + 0.03% of astaxanthin (by weight)	0.03% astaxanthin: ↑ eWAT ↓ Plasma TAG ↓ AST ↓ ALT ↑ HMGR ↑ LDL	0.03% astaxanthin: ↑: ACOX-1, TGF-β1, Nrf2,SOD1, FASN, GCLm, GPx-1 ↓: IL-6

↑: up-regulation; ↓: down-regulation; ACC: acetyl-CoA carboxylase; ACOX-1: acyl-CoA oxidase 1; ACO: acyl-CoA oxidase; ADRP: adipose differentiation-related protein; Anxa1: Annexin A1; ApoB: Apolipoprotein B; ApoE: apolipoprotein E; AST: aspartate aminotransferase; aHSCs: myofibroblasts/activated HSCs; α-SMA: alpha-smooth muscle actin; bFGF: basic fibroblast growth factor; BW: body weight; CAT: catalase; CDAHFD: Choline-deficient, L-amino acid-defined, high-fat diet; CPT-1: carnitine palmitoyltransferase-1; Col1α1: collagen type I alpha 1; Col4α1: Collagen type IV alpha 1; CYP7A1: cholesterol 7α-hydroxylase1; CYP27A1: mitochondrial enzyme sterol 27-hydroxylase; CTGF: Connective tissue growth factor; CPT1α: Carnitine palmitoyltransferase 1 alpha; CD28: Cluster of differentiation 28; CD163: receptor for haptoglobin–haemoglobin complexes; CD206: macrophage mannose receptor; Chrebp: carbohydrate-responsive element-binding protein; Ccr2: C-C chemokine receptor type 2; Cd11c: integrin αX; Chi3l3: eosinophil-related protein; 3: platelet glycoprotein 4; Cfl1: Cofilin 1; Dnm2: dynamin 2; DIO; diet induced obesity; ERN1: Endoplasmic Reticulum to nucleus signalling 1; ERK: extracellular signal-regulated kinase; eWAT: epididymal adipose tissue; FAS: Fas cell surface death receptor; FASN: fatty acid synthase; FATP5: Fatty acid transport protein-5; FGF21: Fibroblast growth factor 21; FOXA: forkhead box-containing transcription factors; HO-1: Heme Oxygenase-1; HIF-1α: Hypoxia-inducible factor 1-alpha; HFD: high fat diet; HMGCR: 3-Hydroxy-3-Methylglutaryl-CoA reductase; GSH: glutathione; GCLm: glutamate–cysteine ligase regulatory subunit; GPx-1: glutathione peroxidase 1; G6pdx: glucose-6-phosphate dehydrogenase X-linked; iNOS: Inducible nitric oxide synthase; IL-1β: Interleukin-1β; IL-6: Interleukin 6; IL-10: Interleukin 10; Il1b: Interleukin 1 beta; IKZF1: Ikaros family zinc finger protein 1; LDL-c: low-density lipoprotein cholesterol; Lxrα: liver X receptor alpha; Mcp1: monocyte chemoattractant protein 1; Mmp9: Matrix metallopeptidase 9; MCDHF: methionine, choline-deficient and high fat; Mgl1: macrophage galactose-type lectin-1; MAP3K8: Mitogen-activated protein kinase 8; Nrf2: nuclear factor erythroid 2–related factor 2; NAS: NAFLD activity score; NEFA: non-esterified fatty acid; NF-κB: Nuclear factor kappa B; OPN: Osteopontin; PAI-1: plasminogen activator inhibitor-1; PNPLA2: patatin-like phospholipase domain-containing protein 2; PPARD: Peroxisome proliferator-activated receptor delta; Pcna: Proliferating cell nuclear antigen; Parp1: Poly(ADP-Ribose) Polymerase 1; PGC-1α: peroxisome proliferator-activated receptor-γ coactivator 1α; PPARγ: Peroxisome proliferator- activated receptor gamma; PPARα: Peroxisome proliferator-activated receptor-alpha; PML: progressive multifocal leukoencephalopathy; p-IRβ/IRβ: phosphorylated-insulin receptor beta; p-Akt/Akt: phosphorylated-protein kinase B; RXRA: Retinoid X receptor alpha; RICTOR: Rapamycin- insensitive companion of mammalian target of rapamycin; SCD-1: stearoyl-CoA desaturase 1; SOD1: superoxide dismutase 1; SOD3: superoxide dismutase 1; Slc25a24: mitochondrial solute carrier family 25 member 24; SREBP: Sterol regulatory element-binding proteins; Scd1: Stearoyl-CoA desaturase-1; SREBP-1c: Sterol regulatory element-binding protein; Srebp1c: Sterol regulatory element-binding protein 1c; TC: total cholesterol; TG: triglycerides; TNC: tenascin-C; TBARs: thiobarbituric acid reactive substances; TGF-β1: transforming growth factor-beta 1; TNF-α: tumour necrosis factor alpha.

**Table 3:** Effects of astaxanthin on NAFLD in cell models.

References	Cell Model	Experimental Design	Effects	Mechanisms
[27]	Human liver cell, L02	Cells were exposed to an FFA mixture of 800 µM oleic acid and palmitic acid for 48 h 5 groups: Control: 5% BSA; FFA: FFA mixture for 48 h; Ax30: 30 uM astaxanthin + FFA; Ax60: 60 uM astaxanthin + FFA; Ax90: 90 uM astaxanthin + FFA (for another 48 h)	↑ Cell survival rate ↓ Mitochondrial ROS ↑ ATP generation ↑ mtDNA copies ↑ ΔΨm ↓ TG	↑: ApoB, ApoE, CPT-1α, FGF21, FOXA, PGC-1α, PPAR- α, Tm6sf2, UCP1, NRF1, TFAM, FGF21, PGC-1α ↓: Bax, Caspase 9, ADRP, CD36, FATP5, SREBP-1c
[34]	Kupffer cells and fatty hepatocyte (isolated from steatotic liver)	3 groups: Control: Kupffer cells or fatty hepatocyte; HR: Kupffer cells or fatty hepatocyte suffered 4 h of hypoxia and 6 h of reoxygenation; HR + astaxanthin: Kupffer cells or fatty hepatocyte treated with astaxanthin before HR	Kupffer cells: ↓ ROS Hepatocytes cell: ↑ Apoptosis	Kupffer cells: ↑: HO-1, Nrf2, HIF-1α, p-mTOR, p-Akt ↓: IL-1β, TNF-α Hepatocytes cell: ↓:Bax, Bc1-2, Caspase-9, Cleaved caspase 9, Caspase-3, Cleaved caspase-3, p-p38 MAPK, p-ERK, p-JNK
[30]	Mouse primary hepatocytes, isolated from a male C57BL/6J mouse	Primary hepatocytes were incubated with either astaxanthin or α-tocopherol for 6 h Treatment: 400 μM oleic acid and astaxanthin (25, 50, 100 μM) for another 16 h	↓ TG ↓ Lipid accumulation	↓ CD36
[40]	Primary HSCs isolated from C57BL/6J mouse liver and LX-2 cells	Primary HSCs: astaxanthin (25 μM) was added at day 2 or day 4 after plating until day 6 LX-2 cells: incubated with astaxanthin (varying concentrations) for 12 or 24 h, and subsequently activated by 2 ng/mL of TGFβ1	LX-2 cells: Minimal cytotoxicity ↓ ROS	LX-2 cells: ↓: TGFβ1, α-SMA, Col1A1, Smad3, Smad7, TβRI, TβRIIPrimary HSCs: ↓: α- SMA

↑: up-regulation; ↓: down-regulation; α-SMA: alpha-smooth muscle actin; ApoB: Apolipoprotein B; ApoE: apolipoprotein E; ADRP: adipose differentiation-related protein; CD36: platelet glycoprotein 4; CPT1α: carnitine palmitoyltransferase 1α; Col1A1: collagen type I alpha 1 chain; FATP5: fatty acid transport protein-5; FGF21: fibroblast growth factor 21; FFA: free fatty acids; FOXA: forkhead box-containing transcription factors; HSCs: hepatic stellate cells; HR: hypoxia and reoxygenation; HO-1: heme oxygenase-1; HIF-1α: hypoxia-inducible factor 1-alpha; IL-1β: interleukin-1 beta; Nrf2: nuclear factor erythroid 2–related factor 2; PGC-1α: peroxisome proliferator-activated receptor-gamma coactivator-1 alpha; PPARα: peroxisome proliferator-activated receptor alpha; p-mTOR: phosphorylated-mammalian target of rapamycin; p-Akt: phosphorylated-protein kinase B; p-p38 MAPK: phosphorylated-p38 mitogen-activated protein kinases; p-ERK: phosphorylated-extracellular signal regulated kinase; p-JNK: phosphorylated-Jun N-terminal kinases; Smad3: SMAD family member 3; Smad7: SMAD family member 7; SREBP-1c: sterol regulatory element-binding transcription factor 1; TGFβ1: transforming growth factor; Tm6sf2: transmembrane 6 superfamily member 2; TNF-α: tumour necrosis factor alpha; UCP1: uncoupling protein 1; NRF1: nuclear respiratory factor 1; TFAM: transcription factor A, mitochondrial; TG: triglycerides; TβRI: transforming growth factor β type I receptor; TβRII: transforming growth factor β type II receptor.

## Data Availability

Not applicable.
